# Inhibition of replication of hepatitis B virus using transcriptional repressors that target the viral DNA

**DOI:** 10.1186/s12879-019-4436-y

**Published:** 2019-09-12

**Authors:** Kristie Bloom, Haajira Kaldine, Toni Cathomen, Claudio Mussolino, Abdullah Ely, Patrick Arbuthnot

**Affiliations:** 1 Wits/SAMRC Antiviral Gene Therapy Research Unit, School of Pathology, Faculty of Health Science, University of the Witwatersrand, Private Bag 3, WITS, Johannesburg, 2050 South Africa; 2 Institute for Transfusion Medicine and Gene Therapy, Medical Center - University of Freiburg, Freiburg, Germany; 3 Faculty of Medicine, University of Freiburg, Freiburg, Germany

**Keywords:** HBV, Transcriptional repressor, TALE, KRAB, DNA methylation

## Abstract

**Background:**

Chronic infection with hepatitis B virus (HBV) is a serious global health problem. Persistence of the virus occurs as a result of stability of the replication intermediate comprising covalently closed circular DNA (cccDNA). Development of drugs that are capable of disabling this cccDNA is vital.

**Methods:**

To investigate an epigenetic approach to inactivating viral DNA, we engineered transcriptional repressors that comprise an HBV DNA-binding domain of transcription activator like effectors (TALEs) and a fused Krüppel Associated Box (KRAB). These repressor TALEs (rTALEs) targeted the viral *surface* open reading frame and were placed under transcription control of constitutively active or liver-specific promoters.

**Results:**

Evaluation in cultured cells and following hydrodynamic injection of mice revealed that the rTALEs significantly inhibited production of markers of HBV replication without evidence of hepatotoxicity. Increased methylation of HBV DNA at CpG island II showed that the rTALEs caused intended epigenetic modification.

**Conclusions:**

Epigenetic modification of HBV DNA is a new and effective means of inactivating the virus in vivo. The approach has therapeutic potential and avoids potentially problematic unintended mutagenesis of gene editing.

**Supplementary information:**

**Supplementary information** accompanies this paper at 10.1186/s12879-019-4436-y.

## Background

Chronic infection with hepatitis B virus (HBV) is a major global cause of mortality and morbidity with particular importance to sub-Saharan Africa [1–4]. Persistence of the hepatotropic virus places infected individuals at high risk for complicating cirrhosis and hepatocellular carcinoma. After infecting liver cells the capsid, which contains viral relaxed circular DNA (rcDNA), is transported to the nucleus. Following release of rcDNA, this nucleic acid is ‘repaired’ to form stable covalently closed circular DNA (cccDNA), which serves as the template for transcription of viral genes and formation of the pregenomic RNA (pgRNA). The pgRNA is reverse transcribed by the viral polymerase to form rcDNA in the nascent HBV particles.

To date immune modulators and direct-acting antivirals, such as interferon (IFN) or nucleoside/nucleotide analogs (NAs) respectively, have been used for management of chronic HBV infection. NAs function by inhibiting reverse transcription of pgRNA, and include entecavir and tenofovir [5–7]. A major shortcoming of licensed therapeutics is that they have little effect on the episomal cccDNA and consequently success of eliminating HBV from infected individuals is low [5]. There are several anti-HBV compounds under development, which have a variety of mechanisms of action. These include inhibitors of virion cellular entry [8] and disruptors of capsid assembly (reviewed in [9]).

Strategies employing gene therapy, which include silencing and editing of HBV sequences, have shown promise (reviewed in [10]). Mutagenic gene editors such as zinc finger nucleases, transcription activator-like effector (TALE) nucleases (TALENs) and clustered regularly interspaced short palindromic repeat (CRISPR) with CRISPR-associated (CAS) have all been engineered to disable HBV genes. Although a promising approach, a concern is that off-target mutagenesis of host sequences may cause serious side-effects. In addition, cleavage of HBV DNA integrants within the host genome may result in chromosomal translocation.

**Fig. 1:**
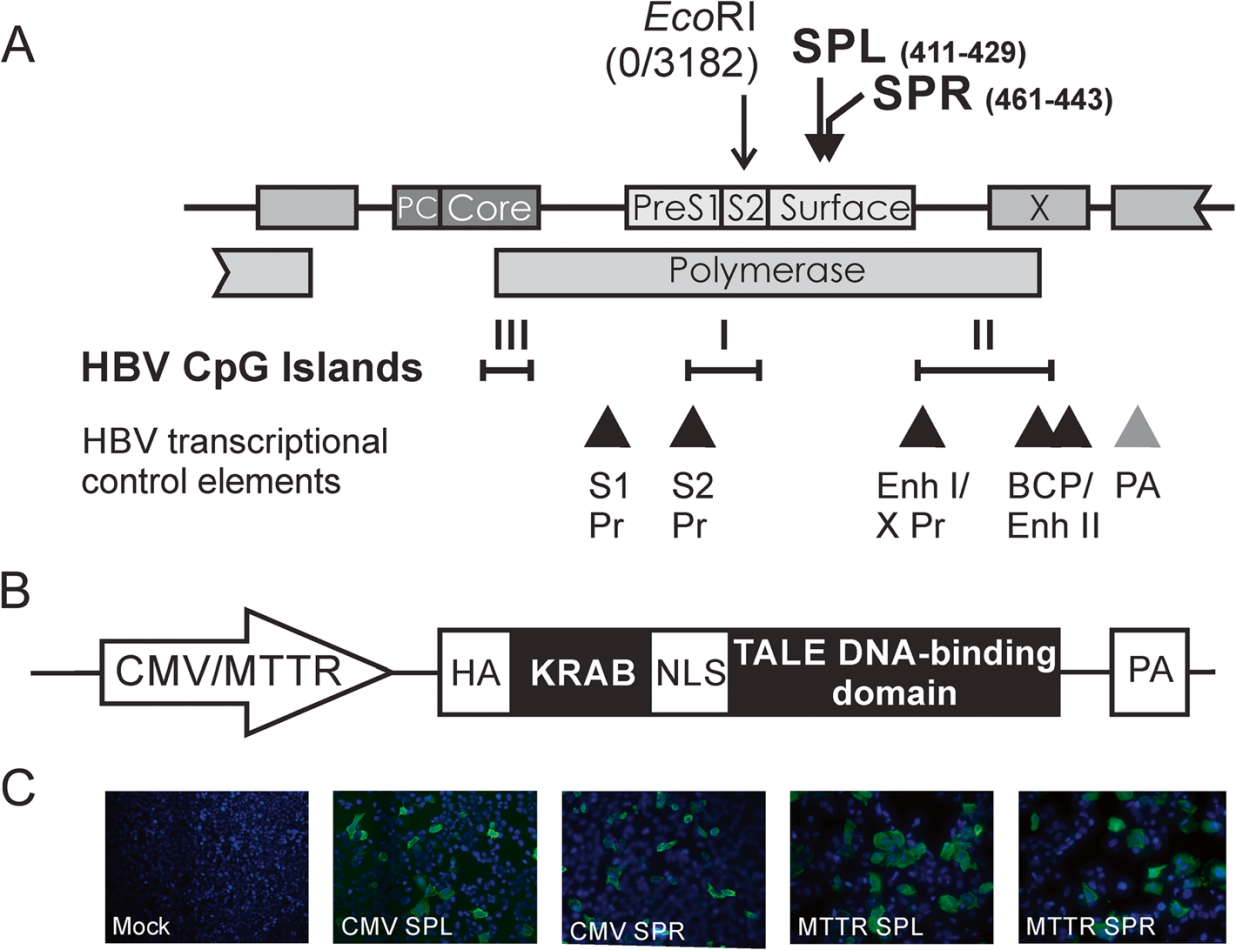
HBV sequence, repressor transcription activator-like effector (rTALE) cassettes and detection of rTALE-coupled hemagglutinin. **a** Open reading frames encoded by the HBV sequences, depicted in a linear arrangement, with target sites of SPL and SPR. Approximate location of CpG islands I, II and III are indicated together with transcriptional control elements. S1 PR: Surface 1 promoter: S2 PR: Pre-S2 Promoter; Enh I/X Pr: Enhancer I and X promoter; BCP/Enh II: Basic core promoter and enhancer II; PA: Polyadenylation signal. **b** Schematic depiction of expression cassettes encoding SPL and SPR rTALEs. A cytomegalovirus (CMV) immediate early promoter/enhancer or modified murine transthyretin receptor (MTTR) regulatory sequence was included to drive transcription of downstream sequences encoding a hemagglutinin tag (HA), Krüppel associated box (KRAB), nuclear localization signal (NLS), DNA-binding TALE and transcriptional termination signal (PA). **c** Representative fields showing immunofluorescence detection of the HA tag with Alexa Fluor 488-labeled antibodies in liver-derived Huh7 cells that had been transfected with plasmids containing CMV-SPL, CMV-SPR, MTTR-SPL or MTTR-SPR expression cassettes, or an irrelevant sequence (Mock)

There is accumulating evidence that epigenetic mechanisms control transcription of HBV cccDNA and play an important role in the clinical course of chronic HBV infection [11–13]. Epigenetic modification of cccDNA with exogenous effectors is thus potentially a useful method to inhibit HBV replication without the risks associated with cleaving target DNA. Endogenous epigenetic modifiers of HBV DNA, which may act on cccDNA-associated proteins or directly on the viral DNA, include histone acetyltransferases/deacetylases (HATs/HDACs) [14], lysine methyltransferases (KMTs) [15] and DNA methyltransferases (DNMTs) [16]. The Krüppel associated box (KRAB) is a well characterized transcriptional repressor [17]. In concert with its co-repressor, KAP-1 (TRIM28), KRAB inhibits gene expression through recruitment of enzymes responsible for histone modification and methylation of CpG islands. HBV cccDNA has three CpG islands that may be methylated to inhibit HBV gene expression: island I, island II and island III (Fig. 1a) [18, 19]. Island I overlaps the *pre-S2* and *surface* sequences, island II covers the enhancer I, enhancer II and basic core promoter regions, and island III is located at the overlapping junction of *core* and *polymerase* open reading frames. Methylation of islands II and III occurs commonly and has been described in HBV chronic carriers with reduced viremias [19–21].

Although data has been presented to show that epigenetic modification of HBV cccDNA significantly influences HBV replication [19–21], evidence for feasibility of using epigenetic modifiers to inhibit HBV replication in vivo is currently limited. Confirming efficacy in animal models of HBV replciation is essential for clinical translation of the technology. In this study, we used the sequence-specific DNA binding domains of previously described TALENs [22]. To repurpose the antiviral elements as epigenetic silencers, sequences encoding the FokI nuclease domain of the TALENs were replaced with DNA encoding a KRAB to generate repressor TALEs (rTALEs). Evaluation in cultured cells and in mice showed highly effective inhibition of markers of viral replication with methylation of target DNA in vivo. Epigenetic modification may thus be a viable line of investigation to develop new therapy for HBV.

## Methods

### Plasmids

To generate SPL and SPR rTALE plasmids, the TALE DNA-binding domains from previously described S TALEN-expression vectors [22] were cloned into the pRK5_HA_KRAB_NLS_TAL vector. This destination plasmid contains in-frame sequences encoding the KRAB repressor immediately upstream of a TALE targeting the rhodopsin gene, together with hemagglutinin (HA) and nuclear localization signal (NLS). The anti-HBV DNA binding domains from the TALEN-expression vectors were excised with *Nhe*I and *Bam*HI and cloned into the corresponding sites in pRK5. The resultant rTALE plasmids encoded repressors targeted sense (SPL) or antisense (SPR) sequences of HBV DNA (Fig. 1a and b). To substitute the cytomegalovirus immediate early promoter/enhancer (CMV) with the liver-specific murine transthyretin receptor (MTTR) promoter [23] a synthetic MTTR sequence (Genscript, NJ, USA) was generated with *Bpi*I type IIS restriction enzyme sites located in flanking sequences upstream and downstream of the MTTR promoter (Additional file 1: Figure S1). The sequence design was such that the *Bpi*I-cleaved promoter would yield overhangs that are compatible with sticky ends generated after digestion of CMV SPL or CMV SPR plasmids with *Not*I and *Pci*I. The MTTR element could then be inserted into the rTALE backbone after removal of the CMV promoter using standard procedures. The full sequence is provided in the Additional file 1. The HBV replication-competent plasmid, pCH-9/3091 [24], and CMV eGFP reporter vector [25] have been described previously.

### Cell culture and transfection

Huh7 liver-derived and HEK293 kidney-derived cells were propagated in Dulbecco’s modified Eagle’s medium (DMEM; BioWhittaker, MD, USA) supplemented with 10% fetal calf serum (FCS) (Thermo Fisher Scientific, MA, USA), 100 U/ml penicillin and 100 μg/ml streptomycin. One day prior to transfection using polyethyleneimine, cells were seeded in 48-, 12- or 6-well plates at a density of 60,000; 120,000 or 240,000 cells per well, respectively. For immunofluorescence staining cells seeded in 48-well plates were transfected with 200 ng of CMV-driven rTALE plasmid, mTTR-driven rTALE plasmid or pTZ57R (mock). For knockdown assays cells seeded in 6-well plates were transfected with 180 ng of pCH-9/3091, 100 ng of pCMV-GFP and 1800 ng of CMV-driven rTALE plasmid, mTTR-driven rTALE plasmid or pTZ57R (mock). To assess the effects of individual TALEN subunits [22] on HBV replication, 200 ng pCH-9/3091, 100 ng pCMV-GFP, 1.6 μg of plasmid encoding left or right surface, or 800 ng of pUC118 were used to transfect cells in each well of a 6-well dish. Fluorescence microscopy was used to detect GFP expression and confirm equivalent transfection efficiencies.

Immunofluorescence was employed to detect the HA epitope using mouse anti-HA primary antibody (Abcam, MA, UK) diluted 1:200 in 1% BSA in PBS with secondary Alexa Fluor 488-labeled goat anti-mouse antibody (Thermo Fisher Scientific, MA, USA). Standard counterstaining with DAPI was used to detect cellular nuclear DNA. HBsAg concentrations were measured using the Monolisa™ HBs Ag ULTRA kit (Bio-Rad, CA, USA) according to previously described procedures [22]. Quantitation of HBV RNA containing *surface* and *core* sequences was carried out using reverse transcriptase quantitative PCR (RT qPCR) [26]. To amplify murine *GAPDH*, HBV *surface* and *core* mRNA, the following primer sets were used: mGAPDH F (5′ TTCACCACCATGGAGAAGGC 3′) and mGAPDH R (5′ GGCATGGACTGTGGTCATGA 3′), HBV Surface F (5′ TGCACCTGTATTCCCATC 3′, HBV coordinates 593–610, Accession LC458430.1) and HBV Surface R (5′ CTGAAAGCCAAACAGTGG 3′, HBV coordinates 734–717), HBV Core F (5′ ACCACCAAATGCCCCTAT 3′, HBV coordinates 2299–2316) and HBV Core R (5′ TTCTGCGAGGCGGCGA 3′, HBV coordinates 2429–2414). Human *GAPDH* mRNA from Huh7 cells was amplified using hGAPDH F (5’GAAGGTGAAGGTCGGAGTC3’) and hGAPDH R (5’GAAGATGGTGATGGGATTTC3’). The MTT cytotoxicity assay was carried out as has been described previously [27].

### Northern blot hybridization

Following transfection as described above, RNA was extracted from Huh7 cells then processed according to standard Northern blotting procedures [28, 29]. ^32^P-labeled oligonucleotides, HBV Surface R and hGAPDH R, were used for detection of *HBV* RNA and *GAPDH* mRNA respectively. Bands were detected using a FLA-7000 Imager (FUJIFILM), and signal intensity then measured using densitometry with ImageJ software [30]. Relative HBV RNA concentrations were calculated as a ratio to GAPDH RNA values.

### Evaluation of rTALE efficacy in mice subjected to hydrodynamic injection

The hydrodynamic injection (HDI) method [31] was employed to determine effects of rTALEs on the markers of HBV replication in vivo. These experiments were carried out on the NMRI strain of mice, which were purchased from the South African National Institute for Communicable Diseases. Procedures were carried out in accordance with protocols approved by the University of the Witwatersrand Animal Ethics Screening Committee. At all times the mice were housed in the specific pathogen free facilities of the Central Animal Services of the University of the Witwatersrand. Mice were kept in cages, fed ad libitum with standard chow and subjected to a 12 h light and 12 h dark cycle. Welfare was regularly monitored by qualified veterinary practitioner. Injected solutions contained 5 μg target DNA (pCH-9/3091) and 5 μg rTALE-encoding plasmid or mock (pUC18). The bolus injectate was administered to six-week-old female mice (weighing 25–30 g) as a saline solution comprising 10% of body weight. To enable analysis of statistically significant effects of the rTALEs, each group of mice included 4 or 5 randomly allocated animals. Serum HBsAg concentration and circulating VPEs per microliter were measured as described [22]. Alanine transaminae (ALT) activity was assayed using an Advia® 1800 Chemistry System (Siemens, NY, USA) at the accredited facilities of the South African National Health Laboratory Service (NHLS, Johannesburg, South Africa). Following conclusion of the murine experimentation, animals were sacrificed using carbon dioxide euthanasia.

### MassArray***®***

Quantitative methylation profiling of intrahepatic HBV DNA was performed by Inqaba Biotech (Pretoria, South Africa) using the EpiTYPER®and MassARRAY®systems (Agena, San Diego, CA, USA). Total HBV DNA was extracted from mouse livers using the Qiagen blood mini kit (Qiagen, Hilden, Germany). EpiDesigner software (Agena) was used to design primers to amplify the HBV CpG island II after bisulphite treatment. Primer sequences were CpGIIF: 5’AGGAAGAGAGGTAATTTTTATTGGTTGGGGTTTG3′ and CpGIIR: CAGTAATACGACTCACTATAGGGAGAAGGCTCATTACTAAAAATCCAAAAATCCTC. Results were calculated as the percentage of methylation at defined CpGs across the viral sequence.

### Statistical analysis

Mean and standard error of the mean (SEM) were calculated for each data set. Two-tailed homoscedastic Student’s t tests were performed using GraphPad Prism version 4.00 (GraphPad software, CA, USA). *P* values of < 0.05 were regarded as statistically significant. For the methylation analysis at specific CpGs, statistical significance was determined using a Mann-Whitney test with a 95% confidence interval (CI). Correlation coefficients were calculated using a Spearman rank correlation (ρ).

## Results

### rTALE design, target sites and expression in transfected liver-derived cultured cells

Monomeric rTALEs were designed to be transcribed and translated from an expression cassette that was driven by the constitutively active CMV or liver-specific MTTR promoter [23] (Fig. 1b). Downstream sequences encoding the rTALE included an in frame hemagglutinin (HA) tag, the KRAB element, a nuclear localization signal (NLS) and TALE domain with specificity for HBV sequences. Cognates of the TALEs were located in the *polymerase* and *surface* ORFs (Fig. 1a), and targeted the viral sense (SPL) or antisense (SPR) strands. Immunodetection of the HA epitope in transfected liver-derived HuH7 cells verified expression of the rTALE-containing sequences (Fig. 1c).

### Inhibition of markers of HBV replication in transfected cells

**Fig. 2:**
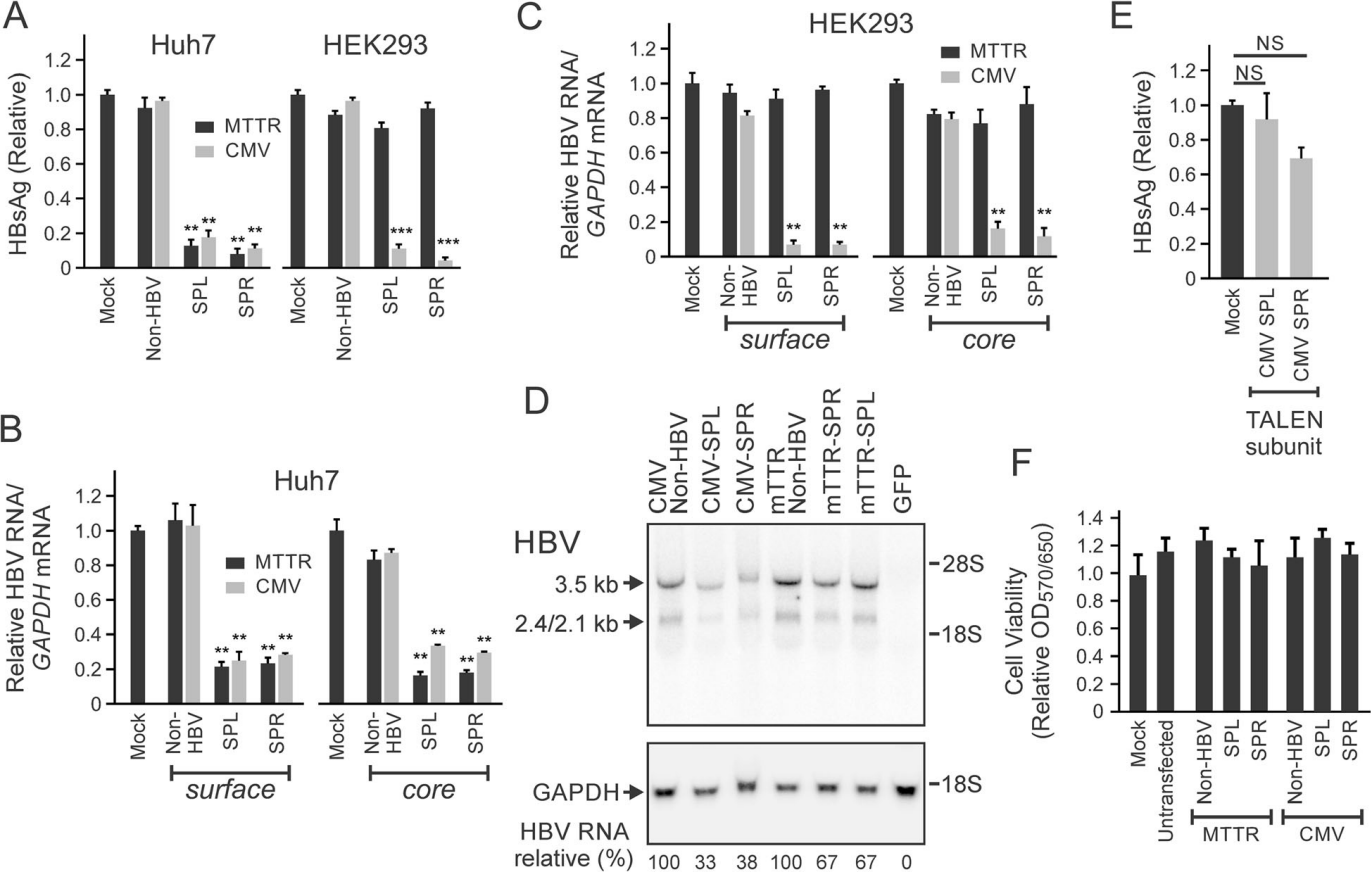
Assessment of rTALE activity and associated toxicity following transfection of cultured cells with DNA encoding rTALEs. **a** HBsAg concentrations in supernatants of cultured liver-derived (Huh7) or non-hepatic (HEK293) cells 48 h after transfection with plasmids encoding SPL and SPR rTALEs from the MTTR or CMV promoter/enhancer elements. Control transfections included an irrelevant DNA sequence (Mock, pTZ57R) or plasmids containing a non-HBV targeting rTALE element. **b** Concentrations of RNA containing HBV *core* and *surface* sequences, isolated from transfected Huh7 cells as described in A, were measured using reverse transcriptase quantitative PCR (RT qPCR). The *glyceraldehyde-3-phosphate dehydrogenase* (*GAPDH*) housekeeping gene was used to normalize the data. **c** Assay of viral RNA as described in B, but after extraction from HEK293 cells. **d** Northern blot analysis of RNA extracted from transfected Huh7 cells. Following resolution on formaldehyde agarose gels, blotted RNA was probed with ^32^P-labeled oligonucleotides complementary to the *surface* sequence of HBV or human *GAPDH*. Ratios of bands’ signal intensities following hybridization to HBV and GAPDH probes were calculated after densitometric scanning. Control cells were transfected with a plasmid encoding Green Fluorescent Protein and did not receive HBV sequences. **e** HBsAg concentrations in supernatants of Huh7 cells 48 h after transfection with plasmids encoding single TALEN subunits (CMV-SPL or CMV-SPR) or pUC118. **f** Assessment of cell viability using the MTT assay in untransfected liver-derived Huh7 cells and following transfection of cells with indicated plasmids as in A. Data are represented as the means and the error bars indicate the standard errors of the mean. All analyses were carried out on a minimum of 3 replicates and data are represented as the mean ± standard errors of the mean (SEM). Statistically significant differences are indicated by asterisks (**: *p* < 0.01; ***: *p* < 0.001)

Plasmids containing CMV- or MTTR-regulated rTALE expression cassettes, together with HBV replication competent and reporter plasmids, were used to co-transfect Huh7 or HEK293 cells. To exclude a non-specific effect of TALEs, control non-HBV plasmids were included which encoded an rTALE that targeted an irrelevant sequence (rhodopsin). Measurement of HBV surface antigen (HBsAg) in the culture supernatants of transfected Huh7 cells revealed inhibition of secretion of the marker of viral replication by both CMV and MTTR promoter cassettes (Fig. 2a). Knockdown was approximately five-fold when compared to the control. When non-hepatic HEK293 cells were transfected, HBsAg concentration was only decreased by DNA encoding the rTALE transcribed from the CMV promoter.

Because the rTALEs have a silencing rather than degrading effect on HBV DNA, we do not expect that concentrations of HBV DNA should be diminished in this short-term experiment. To determine effects of the rTALEs on intracellular viral RNA concentrations, qRT PCR was carried out on extracted RNA. Two separate assays were performed with primers that amplified *core* or *surface* viral sequences. As with the effect on HBsAg secretion, liver-specific expression of the rTALEs from the MTTR promoter correlated with decreased RNA concentrations in Huh7 but not in HEK293 cells (Fig. 2b and c). Northern blot hybridization, carried out on RNA extracted from transfected Huh7 cells, provided more specific information on the effect on *surface* mRNA (Fig. 2d) and confirmed that the rTALE-expressing plasmids diminished concentrations of HBV transcripts. To exclude an effect of DNA-binding regions comprising TALEs without KRAB domains, cells were also transfected with expression cassettes encoding single subunits of previously described effective TALENs [22]. Because TALEN subunits are required to dimerize before duplex DNA can be cleaved and edited, single subunits should be able to bind HBV targets but inhibitory action resulting from target mutation should be minimal if detectable. HBsAg concentrations in the culture supernatants were not significantly affected by individual TALEN subunits (Fig. 2e).

To exclude toxicity of rTALEs as a cause for inhibiting markers of HBV replication in transfected cells, an MTT assay was performed (Fig. 2f). Analysis revealed that there was no significant decrease in cell viability when cells were transfected with the rTALE-encoding plasmids. Based on the antiviral efficacy observed in cultured cells the repressors were then evaluated in vivo.

### Efficacy of rTALEs in vivo

**Fig. 3:**
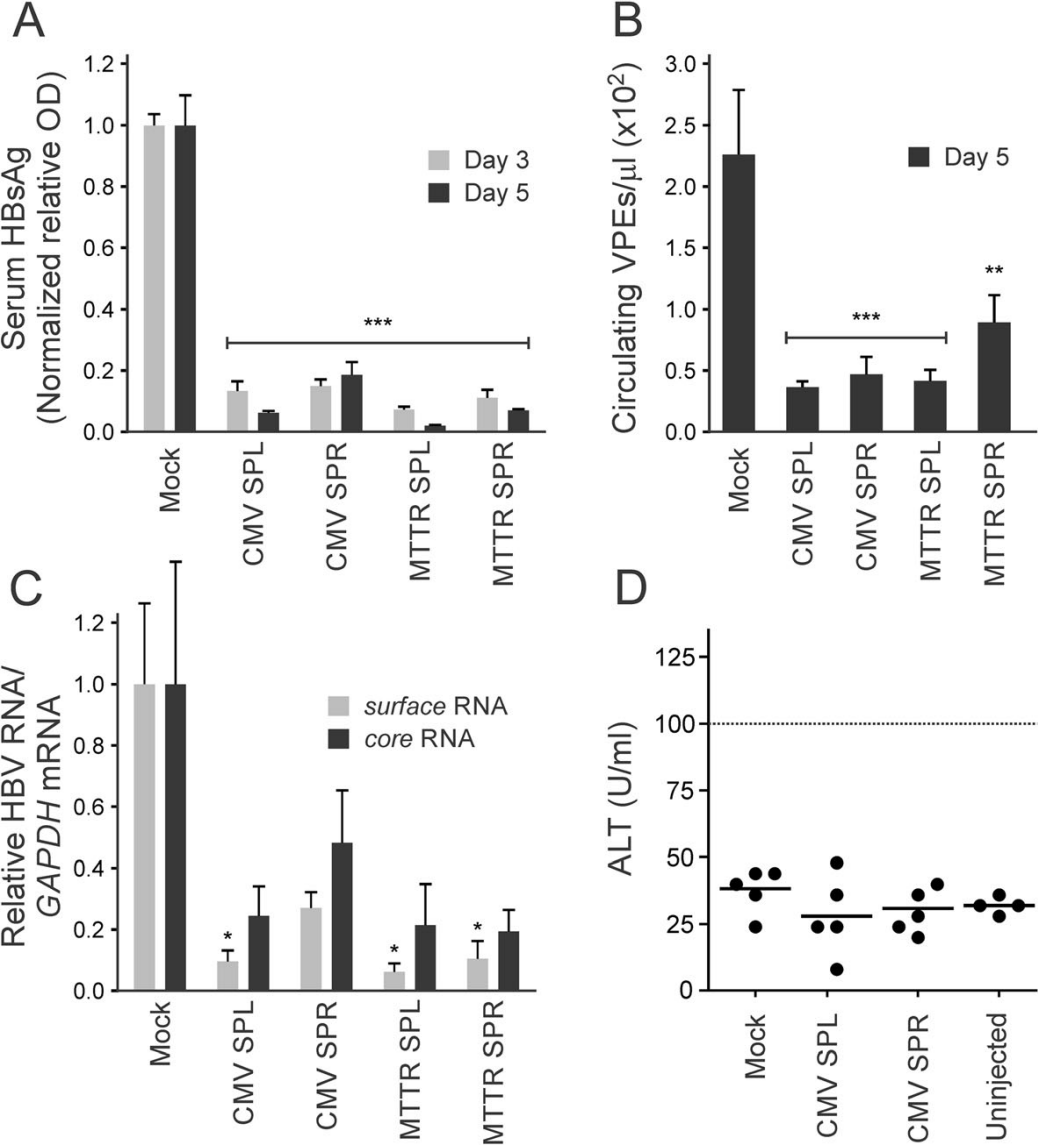
Evaluation of anti-HBV efficacy of rTALEs following murine hydrodynamic injection of plasmids encoding the repressors. **a** Serum concentrations of HBsAg were measured in samples collected at days 3 and 5 after injection of the plasmids. Cassettes encoding the rTALEs contained either the CMV or MTTR promoters. Controls were carried out using a plasmid that contained an irrelevant sequence (Mock, pTZ57R). **b** Circulating viral particle equivalents (VPEs) in the serum that was collected at day 5 after the hydrodynamic injection. **c** Concentrations of RNA containing HBV *core* and *surface* sequences, isolated from liver cells at the termination of the experiment on day 5 were measured using reverse transcriptase quantitative PCR (RT qPCR). Data are presented relative to the concentrations of the murine *GAPDH* housekeeping gene. Each mock group comprised 4 animals and the rTALE-treated groups included 5 mice. Data are represented as the means and the error bars indicate the standard errors of the mean (±SEM). Statistically significant differences, relative to the mock-treated animals, are indicated by asterisks (*: *p* < 0.05, **: p < 0.01 and ***: p < 0.001). **d** Serum alanine transaminase (ALT) activity was determined at day 5 after hydrodynamic injection of mice with CMV SPL or CMV SPR plasmids or pTZ57R (Mock). Uninjected animals were also included as an additional control. The upper limit of normal, 100 IU/ml, is indicated by the dashed line on the graph

To assess whether the rTALEs were effective in vivo, healthy mice were subjected to HDI with an HBV replication-competent vector together with the plasmids encoding the rTALEs under control of the CMV or liver-specific MTTR promoters. Using the HDI procedure, hepatic transfection led to transient replication of HBV and assay of markers of viral proliferation could be used to assess inhibitory effects of the rTALEs in vivo. Over the 5 day period of the analysis, concentrations of HBsAg in serum of mice treated with the SPL and SPR rTALE-encoding plasmids were diminished five- to ten-fold when compared to controls (Fig. 3a). These data were corroborated by values for circulating VPEs on day 5 after HDI (Fig. 3b). Further evidence of inhibition of HBV gene expression was provided by measurement of intrahepatic *surface* and *core* mRNA, which was undertaken at the termination of the experiment on day 5 (Fig. 3c). *Surface* RNA-containing sequences were significantly decreased in mice that had received plasmids containing the CMV SPL, MTTR SPL and MTTR SPR cassettes. Mice that received the CMV SPR expression plasmid showed a 3.6-fold reduction in *surface* RNA-containing sequence. However the lack of statistical significance is most likely to be a result of minor inherent variability of DNA delivery during HDI. Animals tolerated the experiments well and ALT was not elevated in mice receiving DNA encoding the CMV SPL and SPR rTALEs, (Fig. 3d). These data indicate that a toxic effect was not responsible for suppression of HBV RNA concentrations.

### Methylation of viral DNA by rTALEs

**Fig. 4:**
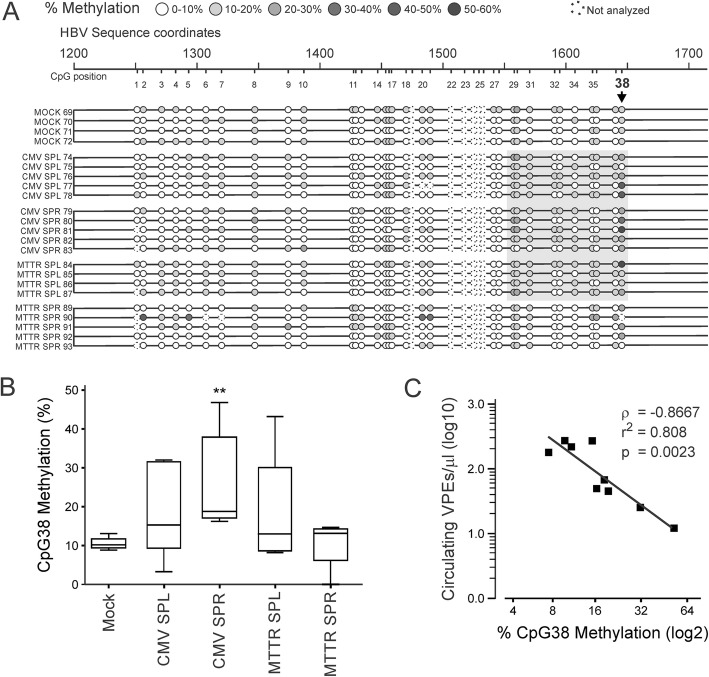
MassARRAY® analysis to evaluate methylation of CpG islands in HBV DNA following hydrodynamic injection of mice. **a** HBV coordinates encompassing CpG island II (see Fig. 1a) are indicated together with each CpG within the sequence. Methylation status of single CpGs was measured from individual mice using MassARRAY® and percent methylation indicated according to the shading. The Mock group of mice (animals numbered 69 to 72) received pTZ57R DNA instead of plasmid with the rTALE cassettes. CMV SPL (animals numbered 74 to 78), CMV SPR (animals 79 to 83), MTTR SPL (animals 84 to 87) and MTTR SPR (animals 89 to 93) received the SPL or SPR rTALE cassette under control of CMV or MTTR promoters. CpGs highlighted by the shaded box indicate where the methylation in the rTALE-treated animals was approximately 50% more than that of the control group. **b** Box and whiskers plot showing the percentage of methylation at CpG38 in mice treated with CMV and MTTR rTALEs. A 2-fold increase was observed for CMV-SPL, CMV-SPR and MTTR-SPL, with a maximum of 51% methylation (error bars = SEM, *n* = 4 for mock and MTTR, *n* = 5 for CMV). *p* = 0.0079 for CMV-SPR using one tailed Mann-Whitney test with confidence interval of 95%. **c** Inverse correlation between the number of circulating VPEs and percentage methylation at CpG38 in control mice and those treated with CMV-SPR rTALE (Spearman correlation coefficient ρ = − 0.8667, r^2^ = 0.808, *p* = 0.0023)

MassARRAY® analysis was carried out to determine the sequence-specific methylation of HBV DNA targets. Importantly, murine hepatocytes do not produce cccDNA [32] and replication-competent pCH-9/3091 plasmid served as the template for this assay. DNA was treated with bisulphite then the HBV sequence encompassing CpG island II was amplified, transcribed and fragmented. Mass spectrometry allowed for efficient evaluation of global methylation status of each CpG within island II from individual mice (Fig. 4a). Methylation was variable across the span of the island. However the region toward the 3’ end of the CpG island was approximately 50% more methylated in mice treated with plasmids containing CMV SPL, CMV SPR and MTTR SPL cassettes. Although some individual mice in the group treated with the MTTR SPR sequences showed increased methylation (e.g. MTTR SPR 90), the effect was inconsistent. Methylation of individual CpGs was therefore carried out to evaluate precise effects of rTALEs (Fig. 4b and Additional file 1: Figure S2). CpG38 from mice treated with CMV-SPR showed statistically significant methylation when compared to controls. Moreover, there was an inverse correlation between the number of circulating VPEs and methylation status of CpG38 (Fig. 4c). Although these data indicate that the rTALEs are inhibiting viral replication as a result of CpG methylation, other epigenetic changes, such as histone modification and methylation at additional CpG islands, may also be contributing to the action of the rTALEs. Action on other novel CpG islands, previously described in different HBV isolates [18], are also possible.

## Discussion

Achieving a functional or complete cure from chronic infection with HBV is a major challenge and is a global focus of current medical research. Eliminating or silencing the viral episomal cccDNA is fundamental to achieving this goal. Several new approaches are being developed to act directly on this viral replication intermediate. Most gene therapy-based work to date has focused on engineered gene editors that are capable of mutating and disabling viral DNA [33]. Clinical studies showing that epigenetic modification of cccDNA influences the course of infected patients is an important consideration in the context of advancing this approach for therapeutic use [19–21]. Our observations that HBV-targeting rTALEs are capable of efficient inhibition of viral replication in cultured cells and in vivo support the notion that this is a feasible therapeutic option. Moreover, the effects were observed without evidence of toxicity.

Several reports have shown that gene therapy has potential for treatment of HBV infection (reviewed in [10]). Most studies have described application of RNA interference (RNAi)-based gene silencing or DNA editing to disable essential viral targets. Harnessing RNAi has entailed use of synthetic formulations containing short interfering RNAs (siRNAs) as well as expressed gene silencers that produce virus-targeting intermediates of the RNAi pathway. Although good efficacy has been demonstrated, a drawback is that inhibition of HBV replication may not be sufficiently durable. Gene editing offers the advantage of causing permanent mutation of viral sequences, which would lead to lasting inhibition of HBV replication. However, it is not clear whether targeted cleavage of integrated HBV DNA would predispose to genotoxic effects such as chromosomal translocation. Moreover, unintended mutation of host cellular sequences resulting from non-specific action of the endonucleases is another possible unintended harmful effect. Efficacy of epigenome editors is thus important and offers advantages over previously described gene therapy-based strategies. Without inducing permanent mutation, rTALEs do not pose the same irreversible risks of virus-targeting endonucleases. However, accomplishing a durable effect of rTALEs and verification of action on cccDNA of patients will be important for therapeutic utility. The stable nature of the epigenetic modification of cccDNA from clinical samples [19–21] suggests that an enduring effect may be likely. Studies involving incorporation of sequences encoding rTALEs into delivery vectors are currently underway and will aid in determining cccDNA-specific antiviral efficacy in pre-clinical models of HBV. Although off-target suppression of host cellular gene expression is a consideration, thorough preclinical evaluation may be realistically achieved. Applying transcriptome sequencing to define mRNA concentrations in treated cells (RNA-Seq) is now a well-established technique and may be applied conveniently to evaluate off-target effects of rTALEs in preclinical models that simulate the human infection.

A dynamic relationship between host and viral factors determines epigenetic regulation and minichromosome formation of cccDNA (reviewed in [34]). HBx promotes viral transcription by controlling histone protein methyltransferase (PMT), DNMT activity, and recruitment of histone modifiers to maintain active replication. KRAB-based repressors may indirectly impede the function of HBx by promoting a heterochromatic state. However the exact mechanism of KRAB repression and assembly of the KAP-1 (TRIM28) co-repressor complex is still unclear [17]. While KAP-1 (TRIM28) recruits a number of pro-methylation proteins (including heterochromatin protein 1 (HP1) isoforms, HDACs, and Setdb1) to facilitate epigenetic modification of target sequences, KAP-1 independent KRAB repression has also been reported [35]. Another study which investigated artificial KRAB repressors based on the CRISPR/Cas9 platform uncharacteristically reported a lack of repressive histone marks at the target effector site, but observed heterochromatin spreading [36]. Indeed KRAB repression has been reported to act up to 25 Kb from the target binding site [37]. For HBV therapy it will be important to characterise which mechanisms are responsible for cccDNA repression and where these epigenetic modifications occur.

## Conclusions

There are several challenges that face gene therapy-based approaches to eliminating HBV, and some are common to advancing epigenome editing for HBV treatment. These include ensuring adequate delivery of therapeutic sequences to sufficient numbers of HBV-infected hepatocytes, guaranteeing specificity of action, and avoidance of innate and adaptive immune responses to the therapeutic. It is likely that progress in the general field of gene therapy will have benefits for HBV therapy. In addition, advances with use of other drugs to treat HBV infection, for example small molecule directly acting antivirals, will be useful if synergistic actions with anti-HBV epigenetic modification of HBV sequences are identified. Our data demonstrating that epigenetic modification may be used to inhibit HBV replication is evidence of a new and effective means of inactivating the virus. This result should have broader applicability and may be useful to treating other viral infections that rely on a stable DNA replication intermediate (e.g. HIV-1).

## Supplementary information

**Additional file 1: Figure S1.** Synthesised sequence encompassing the MTTR promoter. **Figure S2.** Box and whiskers plot of the percentage methylation across CpG points 29 to 38 of HBV island II. (PDF 405 kb)

## Data Availability

The datasets used and/or analysed during the current study are available from the corresponding author on reasonable request.

## Abbreviations

ALT: Alanine transaminase
CAS: CRISPR-associated
cccDNA: covalently closed circular DNA
CI: Confidence interval
CMV: Cytomegalovirus immediate early promoter/enhancer
CRISPR: Clustered regularly interspaced short palindromic repeat
DAPI: 4’,6-diamidino-2-phenylindole
DMEM: Dulbecco’s modified Eagle’s medium
DNMT: DNA methyltransferase
eGFP: enhanced green fluorescent protein
FCS: fetal calf serum
GAPDH: Glyceraldehyde 3-phosphate dehydrogenase
HA: Hemagglutinin
HAT/HDAC: Histone acetyltransferase/deacetylase
HBsAg: HBV surface antigen
HBV: Hepatitis B virus
HDI: Hydrodynamic injection
IFN: Interferon
KRAB: Krüppel Associated Box
MTTR: Murine transthyretin receptor
NLS: Nuclear localization signal
pgRNA: pregenomic RNA
rTALE: repressor TALE
SEM: Standard error of the mean
SPL: rTALE targeting sense surface and polymerase sequences
SPR: rTALE targeting antisense surface and polymerase sequences
TALE: Transcription activator like effector
TALEN: Transcription activator-like effector nuclease
VPE: Viral Particle Equivalent
