# Supplementary material for: Inhibition of replication of hepatitis B virus using transcriptional repressors that target the viral DNA
Source: BMC Infect Dis. 2019 Sep 12;19:802. doi: 10.1186/s12879-019-4436-y (PMC6739920; doi:10.1186/s12879-019-4436-y)
Supplement: Supplementary file 1 — Additional file 1: Figure S1. Synthesised sequence encompassing the MTTR promoter. Figure S2. Box and whiskers plot of the percentage methylation across CpG points 29 to 38 of HBV island II. (PDF 405 kb) [file 12879_2019_4436_MOESM1_ESM.pdf]

*Bpi*I *Pci*I

5’ gcgatgtagaagacg**acatgt**tctttcctgcgttatcccctgattctgtggataaccgtattaccgcctttgagtgagctgataccgctcgccgc
agccgaacgaccgagcgcagcgagtcagtgagcgaggaagcggaagagcgcccaatacgcaaaccgcctctccccgcgcgttgg
ccgattcattaatccagctggcacgacaggtttcccgactggaaagcgggcagtgagcgcaacgcaattaatgtgagttacctcactcatt
aggcaccccaggctttacactttatgcttccggctcgtatgttgtgtggaattgtgagcggataacaatttcacacaggaaacagctatgacc
atgattacgaattaac *tcgagagatctgtcgacaaaattttatcgatcacgagactagcctcgatcgaggtcaattcacgcgagttaataatt*
accagcgcgggccaaataaataatccgcgaggggcaggtgacgtttgcccagcgcgcgctggtaattattaacctcgcgaatattgattc
gaggccgcgattgccgcaatcgcgaggggcaggtgacctttgcccagcgcgcgttcgccccgccccggacggtatcgataagcttagg
agcttgggctgcaggtcgagggcactgggaggatgttgagtaagatggaaaactactgatgacccttgcagagacagagtattaggac
atgtttgaacaggggccgggcgatcagcaggtagctctagaggatccccgtctgtctgcacatttcgtagagcgagtgttccgatactctaa
tctccctaggcaaggttcatatttgtgtaggttacttattctccttttgttgactaagtcaataatcagaatcagcaggtttggagtcagcttggca
gggatcagcagcctgggttggaaggagggggtataaaagccccttcaccaggagaagccgtcacacagatccacaagctcctgccac
catggtgagctagcgatcagg cgcgccatagagtctatggcccaccccccttggcttcgttagaacgcggctacaattaatacataaccttat
gtatcatacacatacgatttaggtgacactatagaataacatccactttgcctttctctccacaggtgtccactcccaggtccaactgcacctc
ggttctatcgattgaattgacatgtacccatacgatgtcccagactacgcgaattta**gcggccgc**gtcttcatcatcgc 3’

*Not*I *Bpi*I

Supplementary Figure 1. Synthesized sequence encompassing the MTTR promoter. The MTTR promoter sequence, together with flanking regions containing *Bpi*I (underlined), *Pci*I (bold) and *Not*I (bold) restriction sites were synthesized. The essential MTTR promoter is indicated in italic font with grey shading. After digestion with *Bpi*I, sticky ends complementary to those of *Pci*I an *Not*I were generated to enable substitution of the CMV promoter with the MTTR promoter in SPL and SPR plasmids.

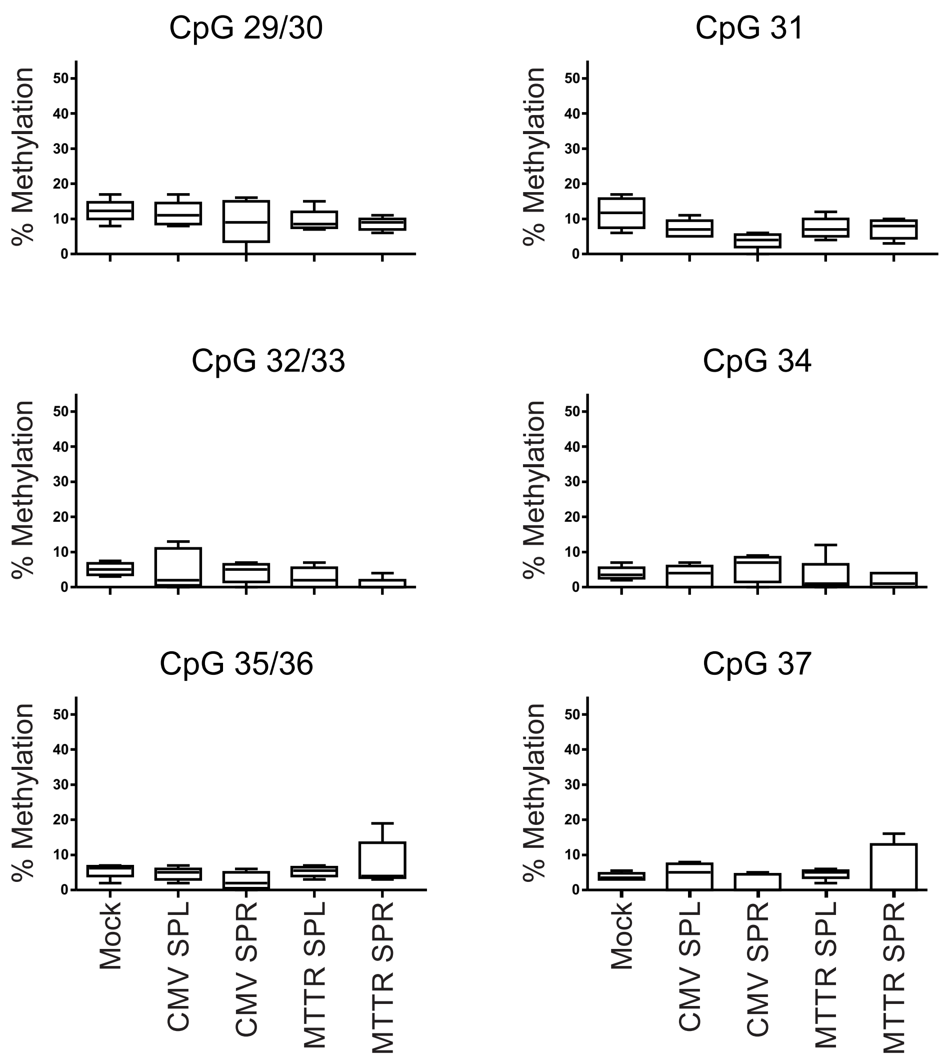

Supplementary Figure 2. Box and whiskers plot of the percentage methylation across CpG points 29 to 38 of HBV island II. Percentage methylation was determined by EpiTYPER MassARRAY on DNA extracted from mouse livers. An increase in the percentage methylation can be seen for MTTR-SPR at CpG positions 35/36 and 37. For CMV-SPL, CMV-SPR and MTTR-SPL, a 2-fold increase in methylation was measured at CpG positon 38.
